# Perivascular fat attenuation index improves the identification of functional myocardial ischemia beyond traditional risk factors: a coronary CT angiography-derived fractional flow reserve study

**DOI:** 10.3389/fcvm.2026.1950032

**Published:** 2026-10-02

**Authors:** Zhihong Gao, Lingmei Jia, Min Jia

**Affiliations:** 1 Department of Health Management Center, The Second Hospital of Hebei Medical University, Shijiazhuang, China; 2 Department of Cardiology, The Second Hospital of Hebei Medical University, Shijiazhuang, China

**Keywords:** coronary computed tomography angiography (CCTA), CT-derived fractional flow reserve (CT-FFR), functional myocardial ischemia, perivascular fat attenuation index (FAI), risk stratification

## Abstract

**Background:**

Perivascular fat attenuation index (FAI) derived from coronary computed tomography angiography (CCTA) is a novel imaging biomarker of coronary inflammation. However, its incremental value in predicting functional myocardial ischemia, defined by CT-derived fractional flow reserve (CT-FFR), beyond traditional cardiovascular risk factors and epicardial adipose tissue (EAT) volume remains unclear.

**Methods:**

A total of 380 consecutive patients who underwent clinically indicated CCTA with CT-FFR evaluation were retrospectively analyzed. Functional ischemia was defined as CT-FFR ≤0.80. Patients were stratified into low and high FAI groups based on the optimal cutoff value of −81.32 Hounsfield Units (HU). Multivariate logistic regression analysis was performed to identify independent predictors of ischemia. The incremental diagnostic performance of adding FAI to a baseline clinical model was assessed using area under the receiver operating characteristic curve (AUC), continuous net reclassification improvement (NRI), integrated discrimination improvement (IDI), and decision curve analysis (DCA).

**Results:**

Despite comparable baseline profiles for low-density lipoprotein cholesterol, body mass index, and glycemic status (*P* > 0.05), patients in the high FAI group exhibited significantly lower CT-FFR values (0.75 ± 0.13 vs. 0.79 ± 0.12, *P* = 0.0017). After adjusting for age, gender, traditional cardiovascular risk factors, and EAT volume, average FAI remained a robust independent predictor of functional ischemia [Adjusted Odds Ratio [OR]: 1.056 per 1 HU increase; 95% Confidence Interval [CI]: 1.019–1.096; *P* = 0.004]. Notably, EAT volume did not reach statistical significance in the multivariate model (*P* = 0.584). The addition of FAI to the clinical model resulted in a modest, non-significant improvement in AUC (0.731 vs. 0.707, *P* = 0.087), but yielded significant improvement in risk reclassification (continuous NRI: 0.339, *P* = 0.001; IDI: 0.024, *P* = 0.002). Subgroup analysis demonstrated a consistent risk trend across varying degrees of stenosis, age, and body mass index categories (OR range: 1.03–1.07). DCA confirmed superior net clinical benefit for the FAI-integrated model across a wide threshold probability range (25%–75%).

**Conclusions:**

Coronary perivascular FAI is an independent imaging marker for CT-FFR-defined functional ischemia and provides incremental value beyond traditional risk factors and EAT volume. These findings support FAI as a non-invasive marker of coronary inflammation that may refine risk stratification for functionally significant coronary artery disease. However, as CT-FFR mainly reflects epicardial physiology, the association between FAI and microvascular dysfunction remains to be determined.

## Introduction

Coronary computed tomography angiography (CCTA) has emerged as a first-line non-invasive diagnostic modality for the assessment of suspected coronary artery disease (CAD), offering high sensitivity and negative predictive value for the exclusion of obstructive stenosis (1, 2). However, the discordance between anatomical stenosis severity and physiological lesion significance is well-documented, particularly in intermediate-grade lesions (30%–75% diameter stenosis) (3, 4). Sole reliance on anatomical assessment may lead to both overutilization of invasive coronary angiography and underestimation of ischemia originating from diffuse non-obstructive atherosclerosis or coronary microvascular dysfunction (5).

The advent of CT-derived fractional flow reserve (CT-FFR) has partially bridged this gap by enabling non-invasive estimation of lesion-specific ischemia using computational fluid dynamics (3). A CT-FFR value ≤0.80 is widely accepted as a surrogate for hemodynamically significant stenosis and guides clinical revascularization decisions (6). Nevertheless, CT-FFR is a “downstream” physiological readout that reflects the cumulative effect of epicardial stenosis, diffuse plaque burden, and microvascular resistance. It does not provide direct insight into the “upstream” biological processes—specifically, vascular inflammation—that drive plaque progression, destabilization, and microvascular functional deterioration (7).

Perivascular adipose tissue is no longer regarded as inert structural support but as a dynamic paracrine organ that exhibits bidirectional crosstalk with the underlying arterial wall (8). In the presence of coronary inflammation, the secretion of pro-inflammatory adipokines inhibits local adipocyte differentiation and lipid accumulation, thereby shifting the perivascular fat composition from a lipid-rich phase to a water-rich phase (9). This phenotypic alteration can be non-invasively captured by measuring the perivascular fat attenuation index (FAI) on routine CCTA images, expressed in Hounsfield Units (HU) (10). Elevated FAI values (less negative HU) have been strongly associated with adverse plaque characteristics, increased cardiovascular mortality, and all-cause mortality in large prospective cohorts such as the CRISP-CT study (11, 12).

However, a critical evidence gap persists regarding the relationship between FAI and functional hemodynamic compromise at the vessel level. While FAI identifies high-risk plaque biology, its incremental value in predicting CT-FFR-defined functional ischemia—beyond traditional cardiovascular risk factors and epicardial adipose tissue (EAT) volume—remains incompletely characterized. Furthermore, it is unclear whether the predictive utility of FAI for ischemia is consistent across key clinical subgroups, such as varying degrees of anatomical stenosis severity, body mass index (BMI) categories, and patient age ranges.

We hypothesized that elevated coronary FAI serves as an independent and robust imaging biomarker of functional myocardial ischemia (CT-FFR ≤ 0.80), providing incremental diagnostic value over conventional clinical models and epicardial fat quantification. In this study, we aimed to: (1) investigate the association between FAI and CT-FFR-defined ischemia; (2) assess the independent predictive power of FAI after rigorous adjustment for confounders including EAT volume; (3) evaluate the incremental clinical utility of FAI-integrated models via net reclassification improvement (NRI) and decision curve analysis (DCA); and (4) explore the consistency of FAI's predictive effect across diverse clinical and anatomical subgroups.

## Methods

### Study design and population

This was a retrospective, single-center observational study. We consecutively screened patients who underwent clinically indicated CCTA with subsequent CT-FFR analysis between January 2024 to December 2025. The study protocol was approved by the Institutional Review Board of The Second Hospital of Hebei Medical University, and the requirement for written informed consent was waived due to the retrospective nature of the analysis.

Inclusion criteria were as follows: (1) age ≥18 years; (2) suspected or known coronary artery disease referred for CCTA; (3) successful CT-FFR computation with evaluable image quality; and (4) complete clinical and laboratory data within one month of the CCTA examination.

Exclusion criteria were: (1) prior history of percutaneous coronary intervention (PCI) or coronary artery bypass grafting (CABG); (2) presence of myocardial bridge or coronary artery anomalies; (3) non-diagnostic CCTA image quality (motion artifacts, severe calcification, or signal-to-noise ratio inadequate for FAI segmentation); (4) acute coronary syndrome at presentation; (5) active malignancy, chronic inflammatory disease, or systemic infection; and (6) missing key variables for FAI or CT-FFR measurement.

A total of 380 eligible patients were ultimately included in the final analysis. Demographic information, cardiovascular risk factors, medication history, vital signs, and standard laboratory parameters were extracted from the electronic medical records. In this study, anatomical coronary artery disease (CAD) was defined as the presence of coronary atherosclerotic plaque on CCTA, whereas severe coronary stenosis was defined as ≥70% diameter stenosis in at least one major epicardial coronary artery. CT-FFR was used to evaluate the hemodynamic significance of coronary lesions and not to establish the anatomical diagnosis of CAD. Angina pectoris was considered present only when angina, stable angina, or unstable angina was explicitly documented in the medical record. Additional functional and invasive examinations, including exercise ECG or stress echocardiography, myocardial perfusion imaging, and invasive coronary angiography, were performed according to clinical indications.

### CCTA image acquisition and reconstruction

All CCTA examinations were performed using a 256-slice CT scanner. Standard acquisition protocols were applied. Patients with a heart rate > 70 beats per minute received oral or intravenous beta-blockade unless contraindicated. Sublingual nitroglycerin (0.5 mg) was administered to all patients prior to scanning to achieve coronary vasodilation.

Prospective electrocardiogram (ECG)-triggered axial scanning or retrospective ECG-gated helical acquisition was employed based on heart rate and rhythm. Images were reconstructed with a slice thickness of 0.5–0.625 mm and an increment of 0.25–0.4 mm. The phase with optimal coronary artery visualization, typically the mid-diastolic phase (70%–75% of the R-R interval) or end-systolic phase (35%–45%), was selected for analysis. A representative example of coronary plaque assessment, CT-FFR analysis, and pericoronary FAI measurement is shown in Figure 1.

**Figure 1:**
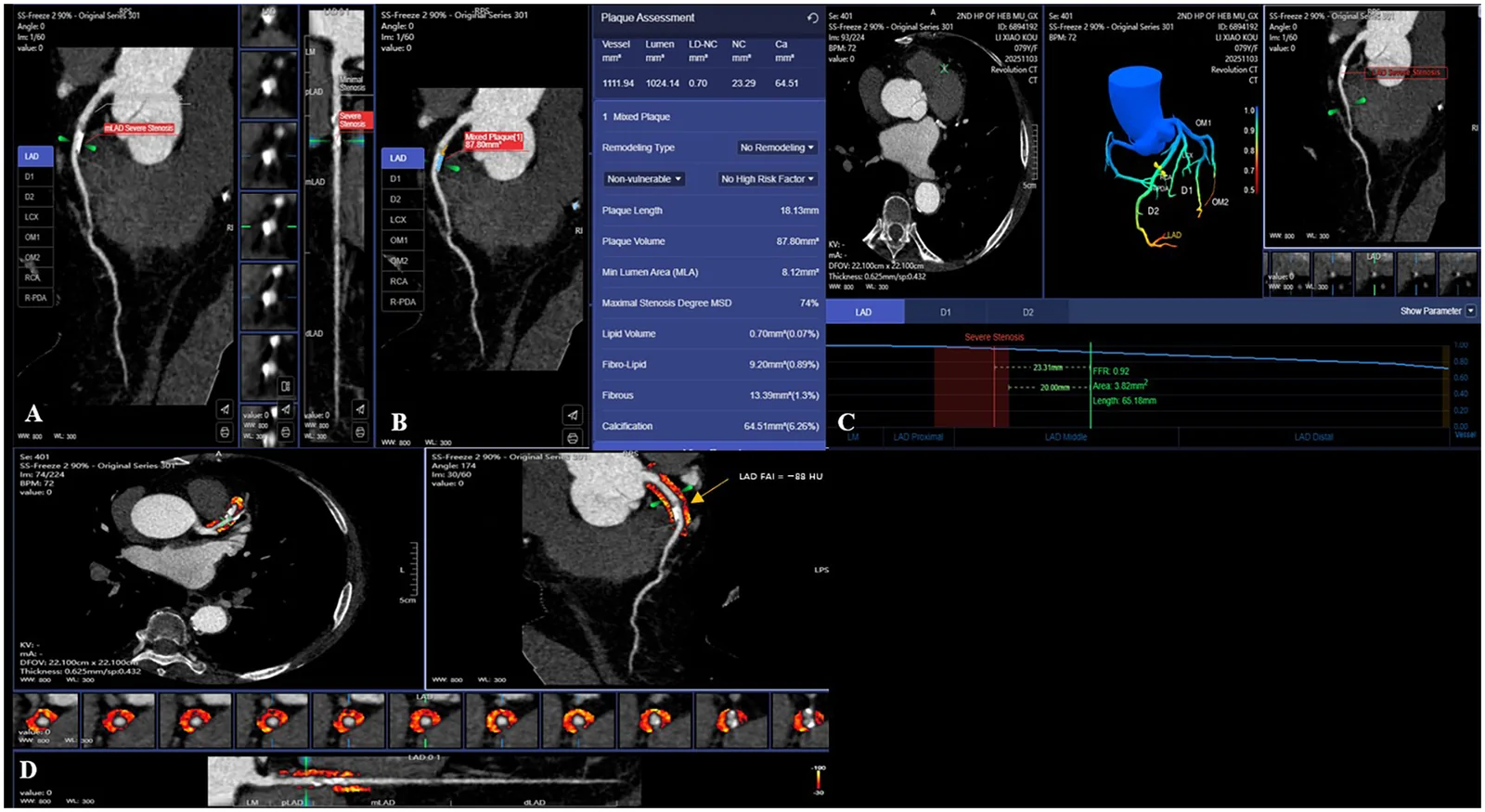
Representative case illustrating coronary plaque assessment, CT-FFR analysis, and FAI measurement. A 78-year-old woman underwent CCTA for evaluation of suspected coronary artery disease. **(A)** Curved planar reformation of the left anterior descending coronary artery (LAD) demonstrates severe stenosis in the mid-LAD, with a maximum diameter stenosis of 74%. **(B)** Automated quantitative plaque analysis demonstrates a predominantly calcified mixed plaque in the LAD, with a total plaque volume of 87.80 mm^3^. **(C)** The color-coded CT-FFR map and longitudinal profile show an LAD CT-FFR value of 0.92. Because this value was greater than 0.80, the lesion did not meet the prespecified criterion for CT-FFR-defined hemodynamic significance. **(D)** Axial, curved planar, and cross-sectional images demonstrate segmentation of pericoronary adipose tissue surrounding the predefined LAD segment. Adipose tissue voxels within the attenuation range of −190 to −30 HU were included in the analysis. The color-coded overlay represents the distribution of pericoronary adipose tissue attenuation, yielding a mean LAD FAI value of −88 HU.

### Perivascular fat attenuation index (FAI) measurement

FAI was automatically quantified using the uAI Research Portal (V20250830; United Imaging Intelligence, Shanghai, China). The methodology adhered to the standardized protocol described by Antonopoulos et al. (13). Briefly, for each major epicardial coronary vessel [right coronary artery [RCA], left anterior descending artery [LAD], and left circumflex artery [LCX]], the proximal 10–50 mm segment was analyzed to avoid the influence of the aortic wall and side branches.

The software automatically segmented the vessel lumen and outer wall boundary. The perivascular adipose tissue was defined as the cylindrical layer of fat extending radially outward from the outer vessel wall to a distance equal to the average vessel diameter. Within this volume of interest, the mean attenuation in Hounsfield Units (HU) was calculated. FAI was defined as the mean attenuation within the perivascular fat volume, with a fixed window range of −190 to −30 HU to exclude fibrous tissue and epicardial structures.

The average FAI (avg_fai) was computed as the arithmetic mean of the FAI values obtained from the three major epicardial vessels (RCA_FAI, LAD_FAI, and LCX_FAI). In the present study, the primary exposure variable was avg_fai per 1 HU increment. This vessel-averaged approach was selected *a priori* to reflect the global burden of perivascular inflammation rather than focal, plaque-specific inflammatory activity, to enhance measurement robustness against single-vessel variability, and to match the patient-level definition of the primary outcome (CT-FFR ≤ 0.80 in at least one major epicardial vessel). Because elevated FAI can also occur in the setting of an unstable plaque confined to a single artery, vessel-specific sensitivity analyses were additionally performed, as described below.

For stratified analyses, patients were dichotomized into low FAI and high FAI groups based on an optimal cutoff value of −81.32 HU, which was derived from receiver operating characteristic (ROC) curve analysis for predicting CT-FFR ≤ 0.80 (maximizing Youden's index). This threshold was data-driven and internally derived from the same cohort; therefore, it should be regarded as exploratory and scanner- and protocol-dependent. External validation in independent cohorts is required before this cutoff can be recommended for clinical application.

### Epicardial adipose tissue (EAT) volume quantification

EAT volume was semi-automatically quantified using the uAI Research Portal (V20250830; United Imaging Intelligence, Shanghai, China). The process involved initial automated segmentation of cardiac tissues, followed by threshold-based segmentation specifically for adipose tissue (ranging from −190 to −30 HU). Finally, the precise boundaries and extent of the epicardial fat were manually adjusted to ensure accuracy.

### CT-derived fractional flow reserve (CT-FFR) computation

CT-FFR was automatically measured using the dedicated post-processing software FFRDoc (V1.0; ShuKun Network Technology, Beijing, China). The software utilizes computational fluid dynamics and coronary physiology models to simulate hyperemic blood flow based on the CCTA anatomic model, with artificial intelligence algorithms similar to those previously validated for CT-FFR computation (14).

After semi-automated lumen segmentation and centerline extraction, the CT-FFR value was computed at every point along the coronary tree. The primary outcome measure was functional ischemia, defined as a CT-FFR value ≤0.80 in at least one major epicardial vessel of ≥2 mm in diameter. The lowest per-vessel CT-FFR value was recorded for each patient. CT-FFR served as the uniform non-invasive reference standard for functional ischemia in this study; other functional tests (e.g., stress electrocardiography, stress echocardiography, and myocardial perfusion imaging) were performed only according to clinical indications and were not used as independent reference standards.

### Statistical analysis

Statistical analyses were performed using R (version 4.5.2) with key packages including rms, pROC, nricens, dcurves, forestplot, and tidyverse. A two-tailed *P* value < 0.05 was considered statistically significant.

Continuous variables were presented as mean ± standard deviation (SD) or median (interquartile range [IQR]), and categorical variables as frequencies (percentages). Normality was assessed using the Shapiro–Wilk test and visual inspection of Q-Q plots. For baseline comparisons between the low FAI and high FAI groups, independent Student's *t*-test, Mann–Whitney *U*-test, Pearson's *χ*^2^-test, or Fisher's exact test were applied as appropriate.

### Prediction model development

Univariate logistic regression analysis was performed to evaluate the association between each candidate variable and functional ischemia (CT-FFR ≤ 0.80). Variables with a *P* value < 0.10 in univariate analysis, along with clinically relevant confounders (age, gender, and EAT volume), were entered into a multivariable logistic regression model using the enter method. Adjusted odds ratios (OR) with corresponding 95% confidence intervals (CI) were calculated.

To assess the incremental diagnostic value of FAI beyond traditional risk factors, we constructed two nested models:

- Model 1 (Clinical Model): Included age, gender, and significant clinical covariates from univariate screening.
- Model 2 (Combined Model): Model 1 + avg_fai.

### Model performance and discrimination

The discriminative ability of the models was evaluated using the area under the receiver operating characteristic (ROC) curve (AUC). DeLong's test was applied to compare AUC differences between nested models. Calibration of the final combined model was assessed using a calibration plot with 1,000 bootstrap resamples to evaluate the agreement between predicted probabilities and observed frequencies.

### Risk reclassification and clinical utility

To quantify the improvement in risk stratification afforded by the addition of FAI, we computed the continuous NRI and the integrated discrimination improvement (IDI). The continuous NRI reflects the overall improvement in risk classification based on predicted probabilities of events and non-events, while the IDI quantifies the average improvement in sensitivity and specificity integrated over all possible risk thresholds. Both continuous and categorical NRI were calculated. Categorical NRI was assessed using prespecified risk categories of <20%, 20%–<50%, and ≥50%.

The clinical net benefit of incorporating FAI into the diagnostic algorithm was assessed using DCA. Net benefit was plotted against a range of threshold probabilities (0%–100%) for defining high-risk ischemia.

### Subgroup and sensitivity analyses

Prespecified subgroup analyses were conducted to explore the consistency of the association between FAI and functional ischemia across clinically relevant strata: age (<65 years vs. ≥65 years), body mass index (BMI < 24 kg/m^2^ vs. ≥24 kg/m^2^), smoking history (non-smoker vs. smoker), and anatomical stenosis severity (30%–75% vs. ≥70% diameter stenosis on CCTA). The effect estimates (OR and 95% CI) for FAI within each subgroup were derived from univariate logistic regression models, and forest plots were generated to visually inspect heterogeneity. A test for interaction was not formally performed due to the exploratory nature of the subgroup analyses.

All analyses were performed on complete-case data. No imputation was performed for missing data given the low rate of missingness (<5%).

As a supportive sensitivity analysis, avg_fai was replaced, one at a time, by LAD_FAI, LCX_FAI, and RCA_FAI in separate multivariable logistic regression models using the same covariate adjustment as the primary model. The outcome remained patient-level CT-FFR-defined ischemia (CT-FFR ≤ 0.80 in at least one major epicardial vessel). Because only the lowest CT-FFR value per patient was retained without its vessel identity, these analyses were not anatomically paired vessel-level comparisons.

## Results

### Baseline characteristics

A total of 380 patients (mean age 63.5 ± 10.7 years; 55.5% male) were included in the final analysis. Among them, 356 (93.7%) had CCTA-defined anatomical CAD, 225 (59.2%) had severe coronary stenosis ≥70%, and 194 (51.1%) had CT-FFR-defined functional ischemia (CT-FFR ≤ 0.80). Clinically documented angina pectoris was present in 175 patients (46.1%). Overall, 237 patients (62.4%) underwent at least one additional functional or invasive examination, including exercise ECG or stress echocardiography in 189 (49.7%), myocardial perfusion imaging in 83 (21.8%), and invasive coronary angiography in 102 (26.8%); because some patients underwent more than one examination, these categories were not mutually exclusive. These data are also presented in Supplementary Table S1.

The optimal cutoff value of average FAI for predicting functional ischemia was determined to be −81.32 HU by maximizing Youden's index. Based on this threshold, 190 patients were classified into the low FAI group (FAI ≤ −81.32 HU) and 190 patients into the high FAI group (FAI > −81.32 HU).

The baseline demographic, clinical, laboratory, and imaging characteristics stratified by FAI group are summarized in Table 1. Notably, traditional cardiovascular risk factors, including age (64.3 ± 10.0 vs. 62.7 ± 11.3 years, *P* = 0.158), body mass index (BMI; 25.7 ± 4.1 vs. 25.9 ± 4.0 kg/m^2^, *P* = 0.582), systolic blood pressure (132.2 ± 16.1 vs. 131.8 ± 17.2 mmHg, *P* = 0.812), low-density lipoprotein cholesterol (LDL-C; 2.5 ± 1.1 vs. 2.4 ± 0.9 mmol/L, *P* = 0.099), fasting blood glucose (5.2 ± 1.9 vs. 5.5 ± 2.1 mmol/L, *P* = 0.231), and the prevalence of hypertension (59% vs. 60%, *P* = 0.917) and diabetes mellitus (17% vs. 22%, *P* = 0.196), did not differ significantly between the two groups.

**Table 1:** Comparison of clinical, laboratory, and imaging characteristics between patients stratified by fat attenuation index.

Variables	All samples (*N* = 380)	Low FAI (≤−81.32 HU)	High FAI (> −81.32 HU)	*P* value
Demographics				
Age (years)	63.50 ± 10.65	64.27 ± 9.95	62.73 ± 11.27	0.158
Gender (Male, %)	—	0.43	0.68	<0.001
Height (cm)	165.84 ± 8.38	164.22 ± 7.86	167.46 ± 8.59	<0.001
Weight (kg)	71.41 ± 12.74	69.86 ± 11.87	72.95 ± 13.37	0.018
BMI (kg/m^2^)	25.83 ± 4.03	25.72 ± 4.08	25.94 ± 3.99	0.582
Clinical History and Blood Pressure				
Systolic blood pressure (mmHg)	132.01 ± 16.62	132.21 ± 16.09	131.80 ± 17.17	0.812
Diastolic pressure (mmHg)	80.38 ± 11.04	79.81 ± 10.93	80.94 ± 11.16	0.322
History of hypertension (%)	—	0.59	0.60	0.917
History of diabetes (%)	—	0.17	0.22	0.196
Smoking history (%)	—	0.17	0.25	0.058
Laboratory Tests				
Total cholesterol (mmol/L)	4.07 ± 1.32	4.17 ± 1.46	3.96 ± 1.15	0.121
Triglyceride (mmol/L)	1.60 ± 3.01	1.50 ± 0.88	1.69 ± 4.17	0.535
Low-density lipoprotein cholesterol (mmol/L)	2.44 ± 1.02	2.53 ± 1.10	2.35 ± 0.93	0.099
Fasting blood glucose (mmol/L)	5.34 ± 2.00	5.21 ± 1.92	5.47 ± 2.08	0.231
White blood cell count (10^9^/L)	6.06 ± 1.84	5.91 ± 1.84	6.21 ± 1.83	0.118
Creatinine (*μ*mol/L)	68.88 ± 37.31	63.95 ± 21.65	73.80 ± 47.61	0.011
Imaging and Functional Studies				
CT-FFR	0.77 ± 0.13	0.79 ± 0.12	0.75 ± 0.13	0.002
Epicardial fat (cm^3^)	144.37 ± 54.14	149.06 ± 54.86	139.68 ± 53.13	0.091
RCA FAI (HU)	−83.81 ± 8.56	−89.16 ± 6.39	−78.45 ± 6.95	< 0.001
Avg FAI (HU)	−80.91 ± 7.21	−86.33 ± 3.57	−75.49 ± 6.19	< 0.001

Continuous variables are presented as mean ± SD; categorical variables are presented as proportions. Patients were stratified into Low FAI (≤ −81.32 HU) and High FAI (> −81.32 HU) groups based on the optimal cutoff of avg_FAI. Between-group comparisons were performed using Student's *t*-test or the Mann–Whitney *U* test for continuous variables, and the chi-square test or Fisher's exact test for categorical variables, as appropriate. “—” indicates data not reported. *P* < 0.05 was considered statistically significant.

FAI, fat attenuation index; BMI, body mass index; SBP, systolic blood pressure; DBP, diastolic blood pressure; HTN, hypertension; DM, diabetes mellitus; TC, total cholesterol; TG, triglycerides; LDL, low-density lipoprotein; FBG, fasting blood glucose; WBC, white blood cell count; Cre, creatinine; CT-FFR, CT-derived fractional flow reserve; EAT, epicardial adipose tissue volume; HU, Hounsfield unit.

However, the high FAI group demonstrated a significantly greater proportion of male patients (68% vs. 43%, *P* < 0.001) and higher serum creatinine levels (73.8 ± 47.6 vs. 64.0 ± 21.7 μmol/L, *P* = 0.011). With respect to imaging parameters, EAT volume trended lower in the high FAI group but did not reach statistical significance (139.7 ± 53.1 vs. 149.1 ± 54.9 cm^3^, *P* = 0.091). Crucially, patients in the high FAI group exhibited significantly lower CT-FFR values compared to those in the low FAI group (0.75 ± 0.13 vs. 0.79 ± 0.12, *P* = 0.0017; Figure 2). Correspondingly, the prevalence of functional ischemia (CT-FFR ≤ 0.80) was substantially higher in the high FAI group than in the low FAI group (59.4% vs. 42.5%).

**Figure 2:**
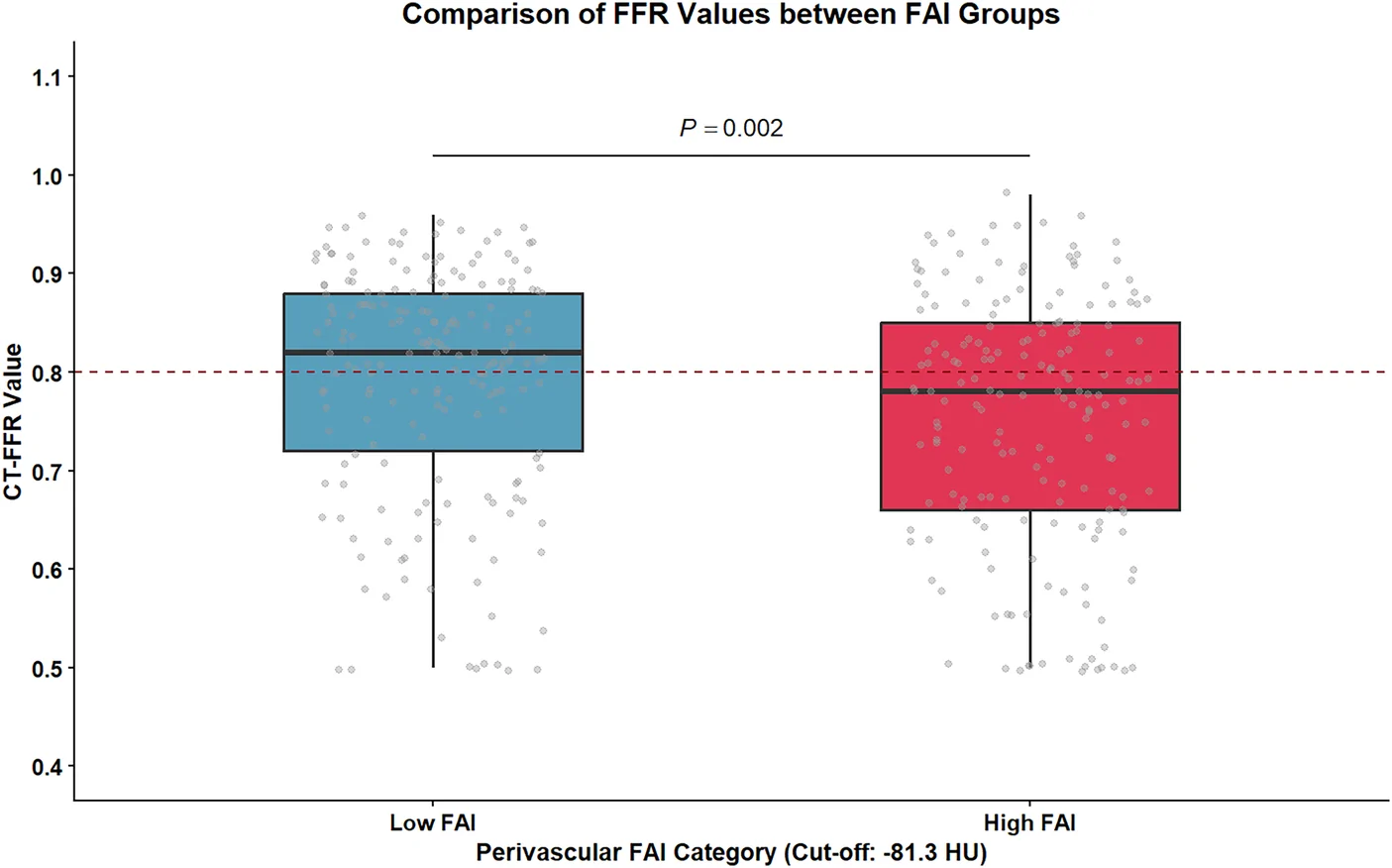
Comparison of CT-FFR values between the low FAI group (FAI ≤ −81.32 HU) and the high FAI group (FAI > −81.32 HU). Box boundaries represent the first and third quartiles, the horizontal line within each box indicates the median, and whiskers extend to the minimum and maximum values excluding outliers. *P* value was derived from independent Student's *t*-test.

### Univariate and multivariate logistic regression analysis

The results of univariate and multivariate logistic regression analyses for the prediction of functional ischemia (CT-FFR ≤ 0.80) are presented in Table 2. In univariate analysis, average FAI (per 1 HU increase) was significantly associated with an increased risk of ischemia [Odds Ratio [OR]: 1.051, 95% Confidence Interval [CI]: 1.015–1.090, *P* = 0.007]. Other variables demonstrating significant univariate associations included age (OR: 1.037 per year, 95% CI: 1.014–1.060, *P* = 0.002), smoking history (OR: 2.239, 95% CI: 1.237–4.200, *P* = 0.009), alcohol consumption (OR: 1.972, 95% CI: 1.068–3.770, *P* = 0.034), hypertension (OR: 1.968, 95% CI: 1.211–3.210, *P* = 0.007), and systolic blood pressure (OR: 1.018 per mmHg, 95% CI: 1.003–1.030, *P* = 0.017).

**Table 2:** Univariable and multivariable logistic regression analysis of factors associated with functional ischemia.

Variables	Univariable analysis		Multivariable analysis	
	OR (95% CI)	*P* Value	Adjusted OR (95% CI)	*P* Value
FAI Indices				
Avg_fai (per HU)	1.051 (1.015–1.090)	0.007	1.056 (1.019–1.096)	0.004
Lad_fai (per HU)	1.012 (0.991–1.040)	0.319	—	—
Lcx_fai (per HU)	1.048 (1.015–1.080)	0.006	—	—
Rca_fai (per HU)	1.052 (1.021–1.080)	0.001	—	—
Demographics				
Age (per year)	1.037 (1.014–1.060)	0.002	1.048 (1.020–1.077)	< 0.001
Gender (Male)	1.547 (0.963–2.500)	0.072	1.240 (0.712–2.164)	0.446
BMI (kg/m^2^)	1.000 (0.944–1.060)	0.987	—	—
Clinical History				
Smoking	2.239 (1.237–4.200)	0.009	1.946 (0.917–4.272)	0.088
Alcohol consumption	1.972 (1.068–3.770)	0.034	1.304 (0.597–2.894)	0.506
Hypertension	1.968 (1.211–3.210)	0.007	1.349 (0.774–2.343)	0.288
Diabetes Mellitus	1.683 (0.935–3.110)	0.088	1.552 (0.820–2.998)	0.182
Hist_hld	1.309 (0.664–2.660)	0.444	—	—
Vital Signs & Laboratory Tests				
SBP (mmHg)	1.018 (1.003–1.030)	0.017	1.015 (0.999–1.031)	0.076
DBP (mmHg)	1.006 (0.985–1.030)	0.559	—	—
HDL-C (mmol/L)	0.584 (0.318–1.020)	0.069	0.589 (0.302–1.104)	0.103
TC (mmol/L)	0.870 (0.723–1.040)	0.128	—	—
TG (mmol/L)	1.042 (0.958–1.300)	0.512	—	—
LDL-C (mmol/L)	0.846 (0.671–1.060)	0.152	—	—
FBG (mmol/L)	0.951 (0.845–1.070)	0.389	—	—
WBC ( × 10^9^/L)	1.110 (0.969–1.280)	0.137	—	—
Neu_abs ( × 10^9^/L)	1.071 (0.928–1.250)	0.360	—	—
Lym_abs ( × 10^9^/L)	0.917 (0.638–1.320)	0.637	—	—
Creatinine (μmol/L)	1.001 (0.995–1.010)	0.800	—	—
Uric acid (μmol/L)	1.001 (0.999–1.000)	0.274	—	—
Imaging				
EAT volume	1.001 (0.997–1.010)	0.584	—	—

Variables with *P* < 0.10 in the univariable analysis or of clinical importance were entered into the multivariable logistic regression model; among the four FAI indices, only avg_fai was retained to avoid multicollinearity. “—” indicates that the variable was not included in the multivariable model.

OR, odds ratio; CI, confidence interval; FAI, fat attenuation index; BMI, body mass index; Hist_hld, history of hyperlipidemia; SBP, systolic blood pressure; DBP, diastolic blood pressure; HDL-C, high-density lipoprotein cholesterol; TC, total cholesterol; TG, triglycerides; LDL-C, low-density lipoprotein cholesterol; FBG, fasting blood glucose; WBC, white blood cell; Neu_abs, absolute neutrophil count; Lym_abs, absolute lymphocyte count; EAT, epicardial adipose tissue.

After adjusting for age, gender, smoking, alcohol consumption, hypertension, diabetes mellitus, systolic blood pressure, high-density lipoprotein cholesterol (HDL-C), and EAT volume in the multivariate model, average FAI remained an independent and robust predictor of functional ischemia (Adjusted OR: 1.056 per 1 HU increase, 95% CI: 1.019–1.096, *P* = 0.004). Age also retained independent predictive value (Adjusted OR: 1.048 per year, 95% CI: 1.020–1.077, *P* < 0.001). Of note, EAT volume was not significantly associated with functional ischemia in the fully adjusted model (*P* = 0.584). Smoking history exhibited a strong trend toward increased risk but did not reach the conventional threshold for statistical significance (Adjusted OR: 1.946, 95% CI: 0.917–4.272, *P* = 0.088).

### Subgroup analysis

To evaluate the consistency of the association between FAI and functional ischemia across clinically important strata, we performed prespecified subgroup analyses. The effect estimates were directionally consistent across all examined subgroups, with ORs ranging narrowly from 1.03 to 1.07 per 1 HU increment in FAI. Similar positive trends were observed across subgroups defined by advanced age, lower BMI, smoking history, and varying degrees of anatomical stenosis, although formal statistical significance was not attained in all strata. As formal interaction testing was not performed, these subgroup findings should be interpreted as exploratory and descriptive rather than as definitive evidence of effect modification (Figure 3).

**Figure 3:**
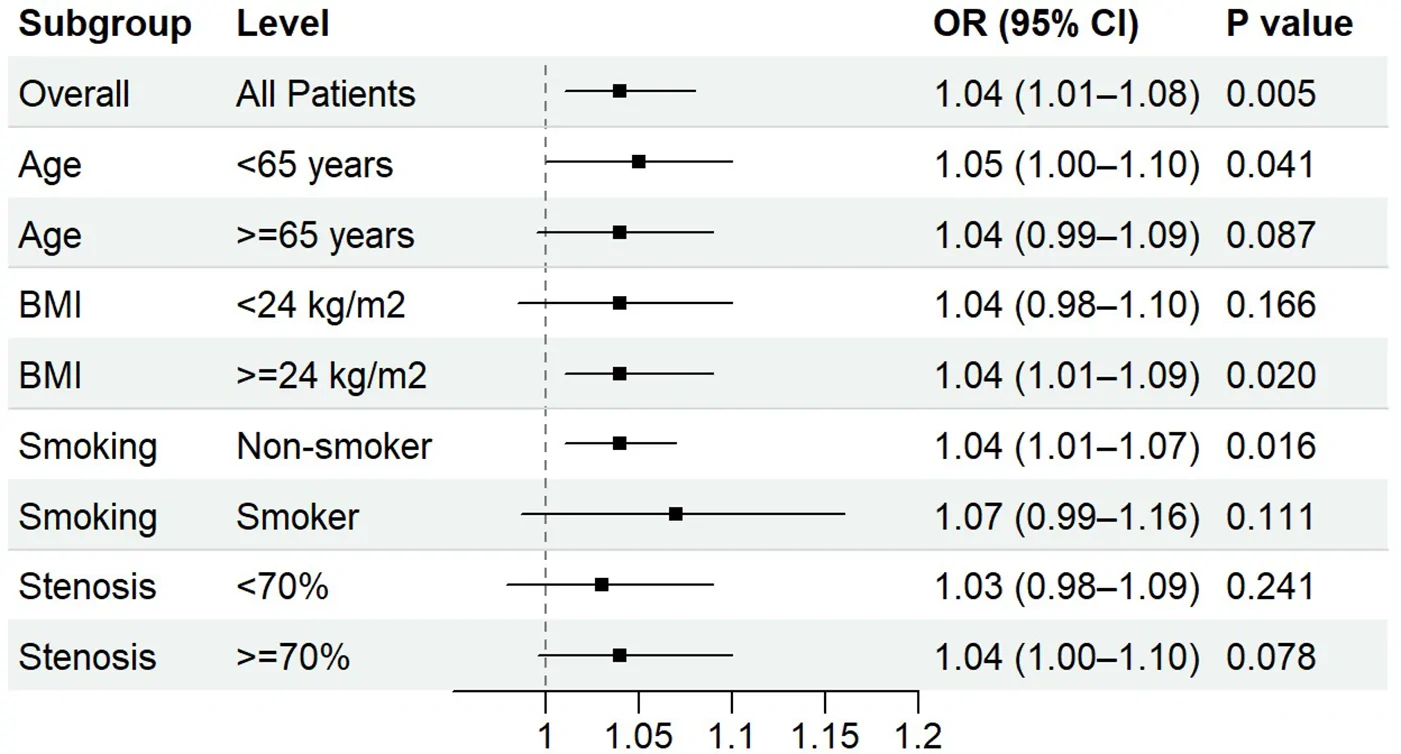
Forest plot demonstrating the consistency of the association between average perivascular FAI (per 1 HU increase) and CT-FFR-defined functional ischemia across prespecified subgroups. Odds ratios and 95% confidence intervals were derived from univariate logistic regression models within each stratum. The dashed vertical line represents an odds ratio of 1.0. *P* values are for within-stratum associations.

In supportive analyses replacing average FAI with individual-coronary FAI measurements, all multiply imputed adjusted models included 380 patients and 194 events. Higher LCX FAI (adjusted OR per 1 HU increase: 1.045, 95% CI: 1.014–1.077; *P* = 0.005) and RCA FAI (adjusted OR: 1.041, 95% CI: 1.014–1.068; *P* = 0.003) remained independently associated with patient-level CT-FFR-defined ischemia. LAD FAI showed a directionally positive but non-significant association (adjusted OR: 1.016, 95% CI: 0.995–1.036; *P* = 0.130) (Supplementary Table S2).

### Incremental diagnostic value of FAI

The diagnostic performance of the clinical model alone, the standalone FAI model, and the combined clinical + FAI model are compared in Figure 4 and detailed in Table 3. The baseline clinical model yielded an AUC of 0.707 (95% CI: 0.644–0.769). The addition of average FAI to the clinical model resulted in a modest, non-significant increase in AUC (0.731 vs. 0.707; *P* = 0.087). Although the improvement in discrimination did not reach statistical significance, the integrated model demonstrated a statistically significant improvement in risk reclassification metrics. The continuous NRI was 0.339 (95% CI: 0.136–0.542, *P* = 0.001), and the IDI was 0.024 (95% CI: 0.009–0.039, *P* = 0.002), indicating improved model discrimination and reclassification. Figure 5 summarizes these metrics and illustrates the shift in predicted probability distributions between the two models.

**Figure 4:**
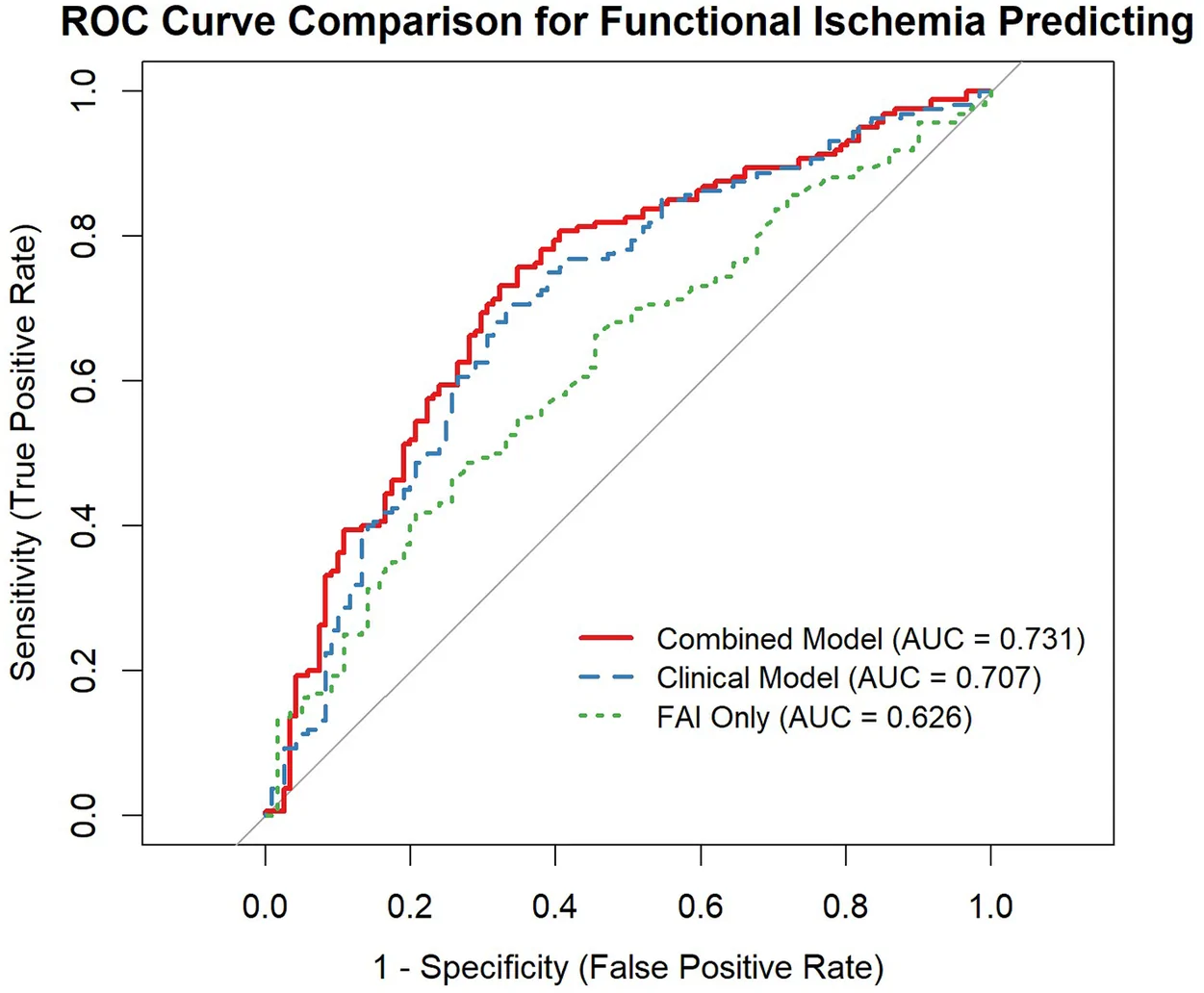
Receiver operating characteristic (ROC) curves for the prediction of functional ischemia (CT-FFR ≤ 0.80). The clinical model (black curve) incorporates age, gender, and traditional cardiovascular risk factors; the standalone FAI model (blue curve) includes average FAI only; the combined model (red curve) integrates FAI into the clinical model. AUC, area under the ROC curve.

**Table 3:** Diagnostic performance of standalone FAI and the combined model for predicting functional ischemia.

Diagnostic model	AUC (95% CI)	Sensitivity	Specificity	*P*-value (vs. clinical)
Clinical Model	0.707 (0.644–0.769)	0.700	0.669	0.687
Standalone FAI	0.626 (0.560–0.691)	0.488	0.727	0.591
Combined Model	0.731 (0.672–0.791)	0.756	0.653	0.712

*P* values compare the AUC of each model with that of the Clinical model using the DeLong test.

FAI, fat attenuation index; AUC, area under the receiver operating characteristic curve; CI, confidence interval.

**Figure 5:**
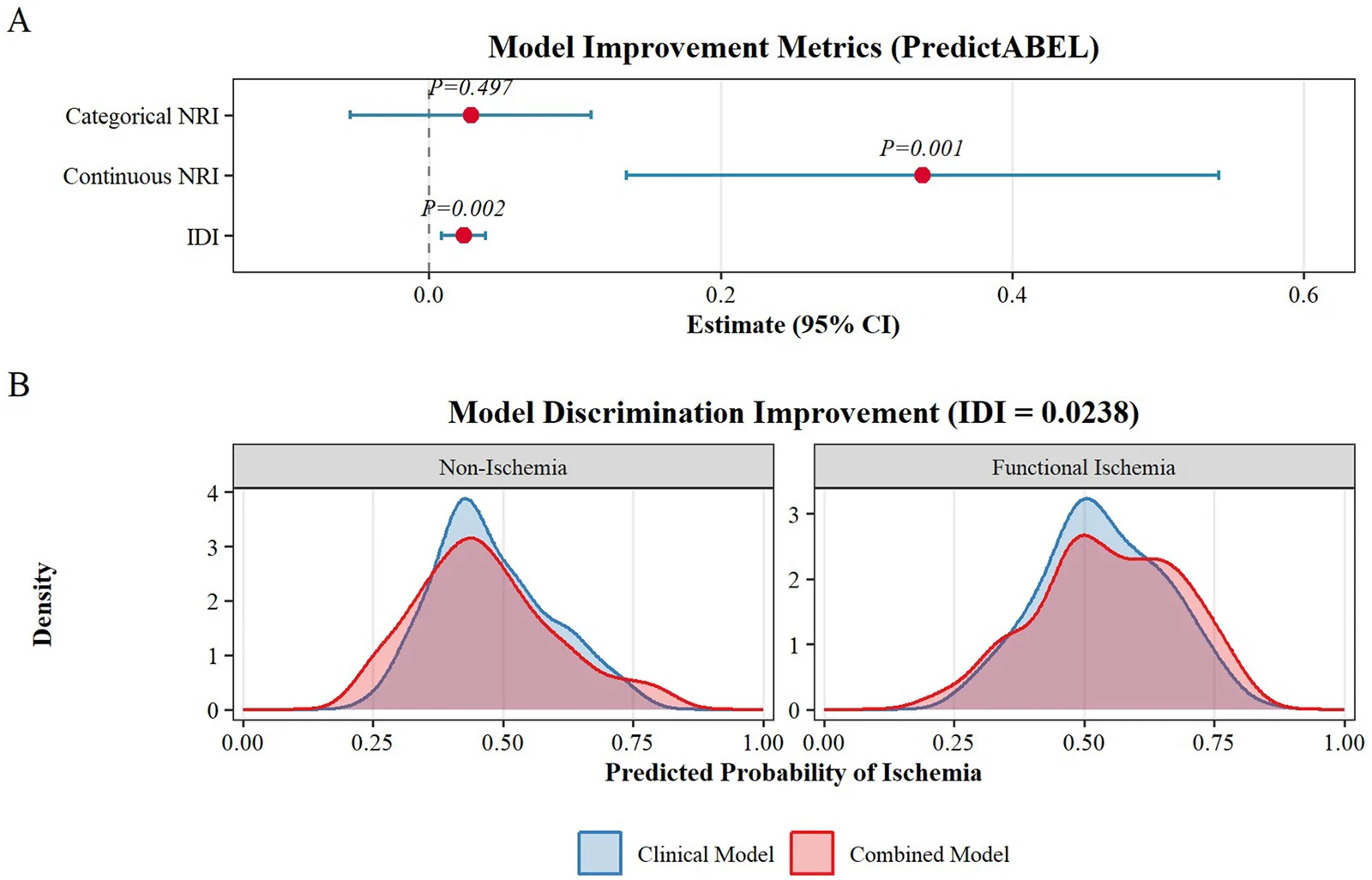
Model improvement metrics after the addition of perivascular FAI to the baseline clinical model. **(A)** Bar plots of continuous NRI and IDI with corresponding 95% confidence intervals and *P* values. **(B)** Density plots of predicted probabilities for patients with and without functional ischemia, illustrating the improvement in discrimination (IDI) between the clinical model (blue) and the combined clinical + FAI model (red).

Restricted cubic spline analysis demonstrated a significant nonlinear association between average FAI and the adjusted odds of functional ischemia (*P* for overall association = 0.0025; *P* for nonlinearity = 0.0223; Figure 6). The odds of functional ischemia increased as FAI values rose above approximately 81.3 HU.

**Figure 6:**
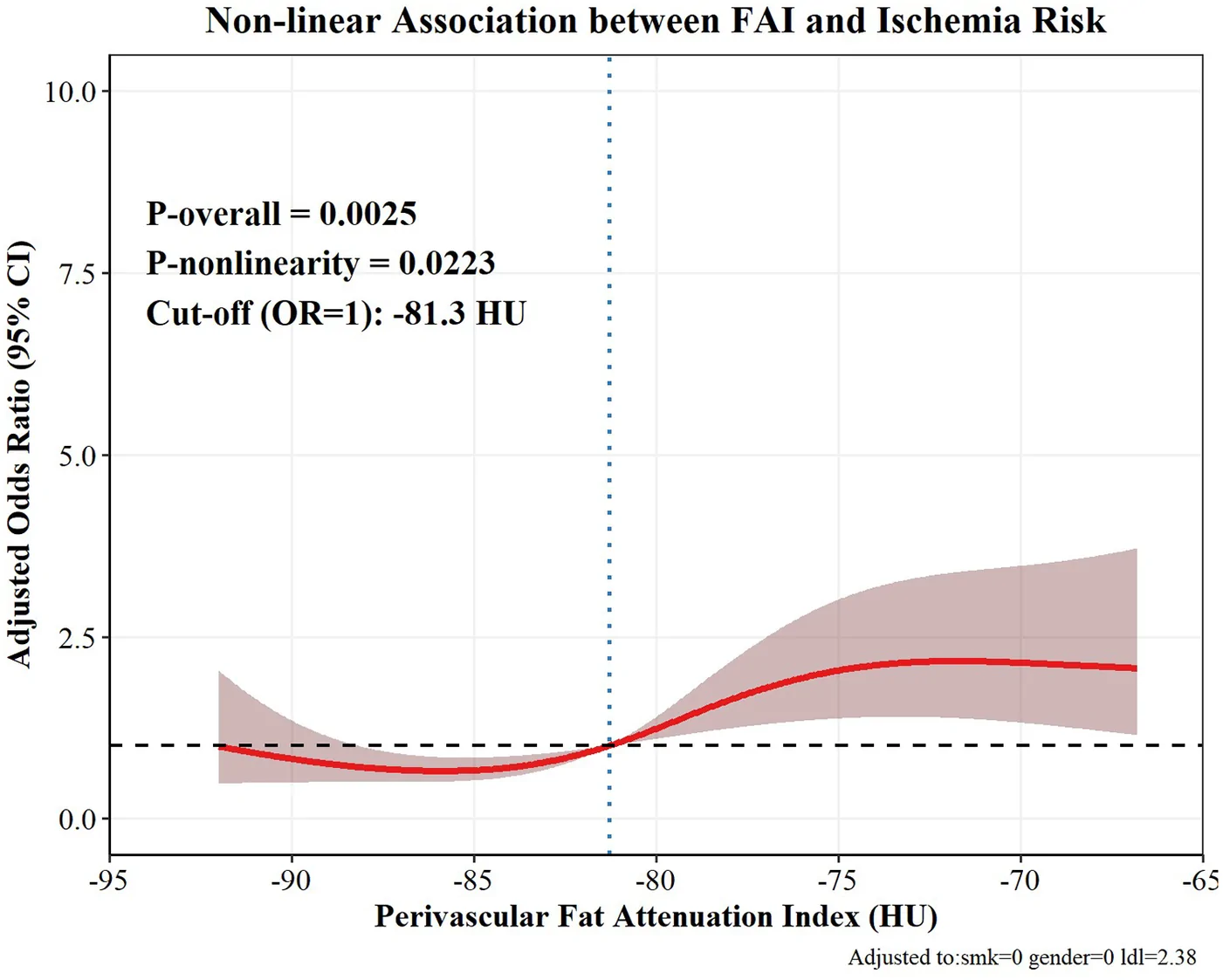
Restricted cubic spline analysis illustrating the dose–response relationship between average perivascular FAI and the adjusted odds ratio for functional ischemia. The solid red line represents the point estimate, and the shaded area denotes the 95% confidence interval. The vertical dashed line marks the optimal cutoff value of −81.32 HU (OR = 1.0). *P* for overall association = 0.002.

A nomogram integrating FAI with clinical variables was constructed to facilitate individualized risk estimation of functional ischemia (Figure 7). The calibration curve for the combined model, derived from 1,000 bootstrap resamples, demonstrated excellent agreement between predicted probabilities and observed frequencies of functional ischemia, with the bias-corrected curve closely approximating the ideal 45° diagonal line, suggesting good calibration and minimal overfitting (Figure 8).

**Figure 7:**
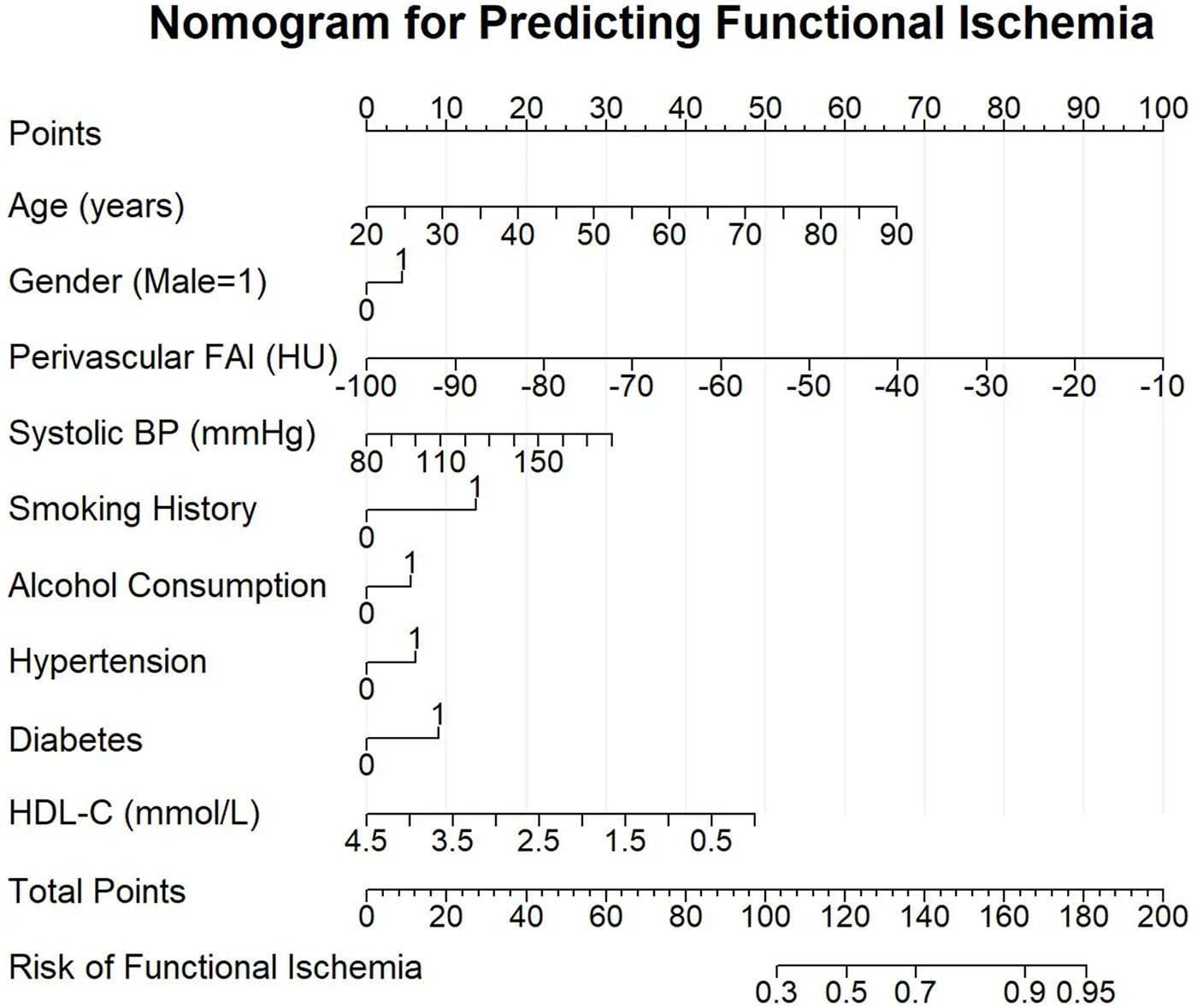
Nomogram for individualized prediction of functional ischemia (CT-FFR ≤ 0.80), integrating age, gender, perivascular FAI, systolic blood pressure, smoking history, alcohol consumption, hypertension, diabetes mellitus, and high-density lipoprotein cholesterol. To use the nomogram, locate each variable on its axis, draw a vertical line to the “Points” scale to obtain the corresponding score, sum all scores to obtain “Total Points,” and read the predicted risk on the bottom axis.

**Figure 8:**
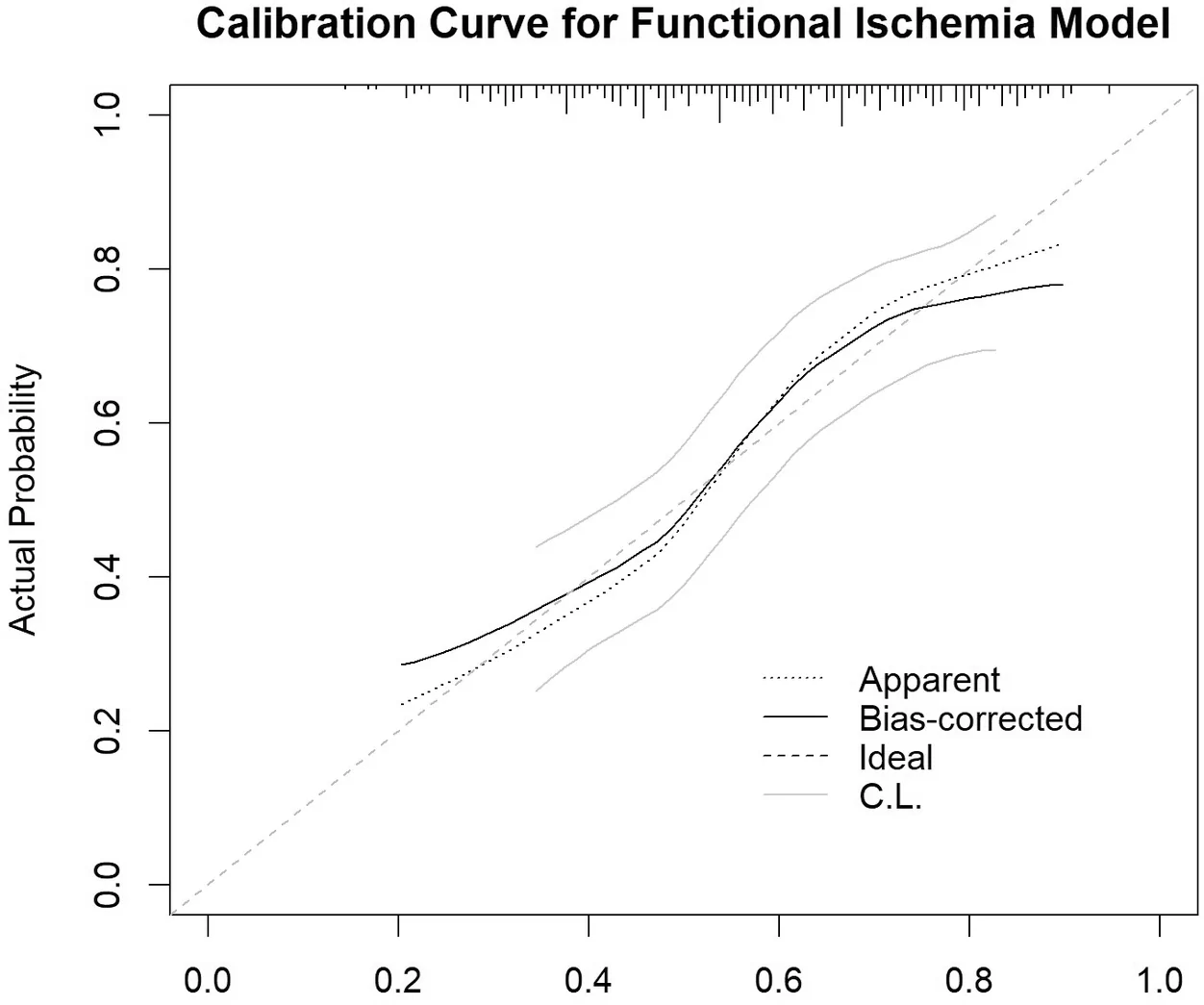
Calibration plot of the combined clinical + FAI model for predicting functional ischemia. The apparent curve (solid line) represents the calibration of the original model; the bias-corrected curve (dashed line) was derived from 1,000 bootstrap resamples. The ideal 45° diagonal line (dotted) indicates perfect agreement between predicted probabilities and observed frequencies.

### Clinical decision curve analysis

The clinical utility of incorporating FAI into the diagnostic algorithm was assessed using DCA (Figure 9). Across a wide and clinically relevant range of threshold probabilities for defining high-risk ischemia (approximately 25% to 75%), the combined clinical + FAI model yielded a consistently higher net benefit compared to both the clinical model alone and the extreme strategies of “treat all” or “treat none.” This finding indicates that the integration of FAI enhances clinical decision-making by improving the identification of patients who would benefit from further investigation or intervention while minimizing unnecessary procedures.

**Figure 9:**
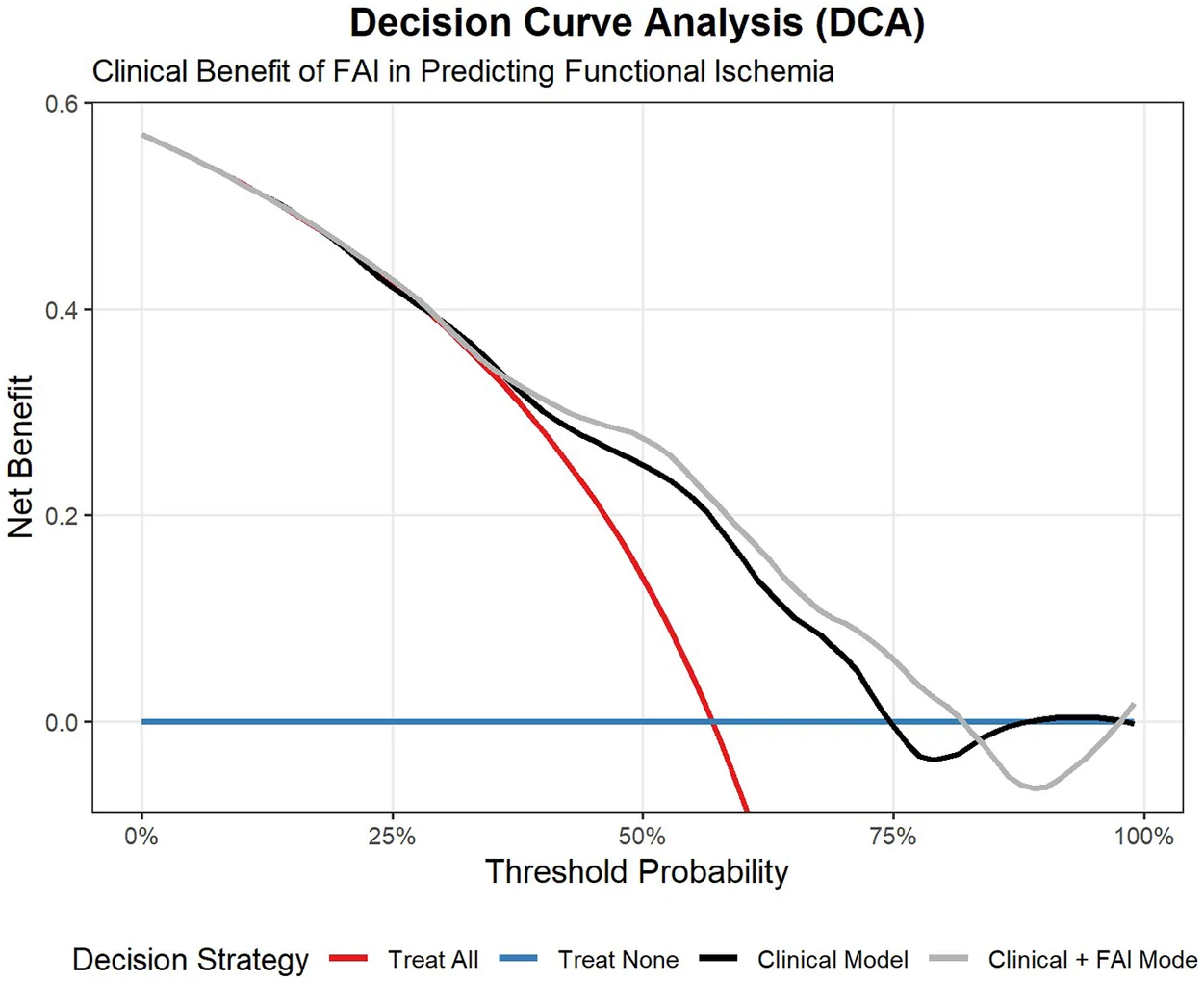
DCA comparing the net clinical benefit of four strategies for identifying functional ischemia across a range of threshold probabilities: treat all patients (red line), treat none (blue horizontal line), clinical model alone (black curve), and combined clinical + FAI model (gray curve). The combined model yields a higher net benefit than the clinical model across the threshold probability range of approximately 25% to 75%.

## Discussion

The present study provides a comprehensive evaluation of the association between coronary perivascular FAI and CT-FFR-defined functional ischemia. Our principal findings are fourfold. First, patients with elevated FAI (>−81.32 HU) exhibited significantly lower CT-FFR values despite comparable profiles of traditional cardiometabolic risk factors, indicating a distinct contribution of perivascular inflammation to coronary physiological impairment. Second, after rigorous adjustment for confounders—including age, sex, traditional risk factors, and notably, EAT volume—average FAI remained an independent predictor of functional ischemia, with each 1 HU increment conferring a 5.6% increased odds of CT-FFR ≤ 0.80. Third, the addition of FAI to a conventional clinical model resulted in a significant improvement in risk reclassification, as evidenced by a continuous NRI of 33.9% (*P* = 0.001) and an IDI of 0.024 (*P* = 0.002). Fourth, the predictive effect of FAI was directionally consistent across diverse clinical and anatomical subgroups, underscoring its robustness as a non-invasive imaging biomarker. Collectively, these observations support the notion that FAI captures a distinct facet of coronary pathophysiology—namely, perivascular inflammation—that is not fully reflected by conventional risk metrics or by volumetric quantification of epicardial adiposity.

### Perivascular inflammation and coronary physiology: linking biology to hemodynamics

The observed inverse association between FAI and CT-FFR aligns with the emerging paradigm that vascular inflammation plays a pivotal role in modulating coronary vasomotor function. Elevated FAI, as measured by CCTA, reflects a phenotypic shift in perivascular adipose tissue from a lipid-laden to a water-rich, pro-inflammatory state, driven by the paracrine release of adipokines in response to underlying arterial wall inflammation (15). This local inflammatory milieu has been shown to impair endothelium-dependent vasodilation and augment microvascular resistance, thereby reducing myocardial blood flow reserve (16). Consistent with this paradigm, recent advances in advanced CT phenotyping have further demonstrated that pericoronary inflammation is closely linked to plaque vulnerability and adverse coronary remodeling, reinforcing the biological plausibility of FAI as a functional imaging biomarker (17).

In our cohort, the high FAI group demonstrated a mean CT-FFR of 0.75, which is widely regarded as a definitive threshold for hemodynamically significant ischemia, compared with 0.79 in the low FAI group (*P* = 0.0017). Importantly, this difference was observed in the context of balanced traditional risk factors—including LDL cholesterol, glycemic status, and BMI—suggesting that perivascular inflammation exerts a direct, deleterious effect on coronary physiological function that operates independently of systemic metabolic dysregulation.

Furthermore, although CT-FFR primarily reflects epicardial coronary physiology and cannot directly diagnose coronary microvascular dysfunction, our findings provide indirect support for the hypothesis that perivascular inflammation may contribute to functional ischemia beyond focal stenosis alone. While epicardial stenosis severity influences CT-FFR values, the persistent association of FAI with ischemia across varying degrees of anatomical narrowing suggests that a component of the observed physiological impairment may involve diffuse or microvascular mechanisms, although this cannot be established definitively from the present data. This interpretation is consistent with prior invasive studies reporting associations between elevated FAI and impaired coronary flow reserve (18).

We also wish to clarify the interpretation of the CT-FFR values reported in this study. All primary analyses—including multivariable logistic regression, ROC curve analysis, risk reclassification (NRI/IDI), subgroup, and decision curve analyses—were based on the binary threshold of CT-FFR ≤ 0.80, consistent with clinical practice and prior validation studies. The comparison of mean CT-FFR values between the low and high FAI groups (0.79 ± 0.12 vs. 0.75 ± 0.13, *P* = 0.0017) was intended only as a descriptive baseline characteristic, illustrating that higher FAI was associated with a leftward shift of the overall CT-FFR distribution; this was corroborated by the significantly higher prevalence of functional ischemia in the high FAI group at the threshold level (59.4% vs. 42.5%). In addition, restricted cubic spline analysis demonstrated a significant nonlinear association between average FAI and the probability of functional ischemia (*P* for overall association = 0.002), supporting a graded association between perivascular inflammation and hemodynamic compromise. We acknowledge that the relationship between small differences in continuous CT-FFR values and the severity of myocardial ischemia remains incompletely characterized, and assessment of ischemic burden at the vessel or territory level was beyond the scope of this study; these questions warrant dedicated investigation.

### Average FAI vs. vessel-specific FAI: does averaging dilute focal inflammatory signals?

One methodological aspect of the present study warrants specific consideration: the use of the average FAI across the three major epicardial coronary arteries as the primary exposure. Because elevated FAI can occur in the presence of an unstable plaque confined to a single artery (15), averaging across vessels could, in theory, dilute a focal inflammatory signal and thereby attenuate the observed associations. Several lines of evidence suggest that this did not materially affect our conclusions. First, the vessel-averaged FAI reflects the global coronary inflammatory burden and is aligned with the patient-level definition of functional ischemia (CT-FFR ≤ .80 in at least one major epicardial vessel), thereby reducing the risk of exposure–outcome misalignment. Second, in vessel-specific sensitivity analyses, replacing the average FAI with RCA_FAI or LCX_FAI yielded independent and statistically significant associations with patient-level ischemia (adjusted OR 1.041 and 1.045 per 1 HU increase, respectively), whereas LAD_FAI showed a directionally consistent but non-significant association (adjusted OR: 1.016, 95% CI: 0.995–1.036; *P* = 0.130). The concordant direction of the effect across all three vessels argues against averaging-induced bias as the primary driver of our findings. Third, the non-significant association observed for LAD FAI most plausibly reflects the anatomic proximity of the proximal LAD segment to the aortic root and major side branches, where perivascular fat measurements may be more susceptible to contamination by adjacent structures, as well as reduced statistical power, rather than a true biological null effect. Nevertheless, our vessel-specific analyses were not anatomically paired comparisons with the ischemia-defining vessel, and future vessel-matched studies linking vessel-specific FAI to ischemia in the corresponding coronary territory are warranted to further refine the clinical application of FAI.

### FAI vs. EAT volume: the primacy of Fat “quality” over “quantity”

A noteworthy finding of our multivariate analysis was the lack of a significant association between EAT volume and functional ischemia in the fully adjusted model (*P* = 0.584). In contrast, FAI—which captures the biological quality rather than the anatomic quantity of perivascular fat—remained a robust predictor. This discrepancy underscores a critical pathophysiological distinction. EAT volume represents the cumulative burden of visceral adiposity surrounding the heart and is a well-established marker of long-term metabolic risk. However, its relationship with functional ischemia at the vessel level may be diluted by the substantial inter-individual variation in thoracic adiposity and by the fact that not all epicardial fat is equally inflamed. FAI, by quantifying the attenuation gradient within the immediate vicinity of the coronary arterial wall, provides a focused assessment of the bidirectional inflammatory crosstalk that directly impacts vascular tone and endothelial health (19). Our data therefore reinforce the emerging concept that “sick fat” (dysfunctional, inflamed adipose tissue) is more relevant to acute physiological compromise than “fat mass” alone. This finding has direct clinical implications: routine volumetric quantification of EAT may be insufficient for risk stratification, and dedicated perivascular fat phenotyping via FAI offers incremental, actionable information.

### Subgroup heterogeneity: age and BMI as modifiers of predictive utility

Our subgroup analysis revealed several patterns of clinical interest. The association between FAI and functional ischemia appeared more pronounced and attained statistical significance in younger patients (<65 years) and in those with higher BMI (≥24 kg/m^2^). In older individuals (≥65 years), the OR for FAI was comparable (1.04), yet the *P* value (0.087) fell short of significance. This may reflect the increasing prevalence of age-related vascular remodeling, medial calcification, and established epicardial plaque burden, which collectively may obscure the relative contribution of active inflammation to the ischemic threshold. In younger patients, whose coronary tree is less encumbered by chronic structural disease, FAI may serve as a more sensitive indicator of early, subclinical inflammatory activity—a hypothesis with potential implications for primary prevention strategies.

The robust performance of FAI in the overweight/obese subgroup (BMI ≥ 24 kg/m^2^, *P* = 0.020) is particularly noteworthy. In individuals with higher adiposity, the perivascular fat depot is typically larger and metabolically more active, potentially providing a stronger signal-to-noise ratio for attenuation-based measurements. This finding supports the clinical utility of FAI in precisely the population that bears a disproportionate burden of cardiometabolic disease and in whom CCTA is frequently indicated.

Regarding anatomical stenosis severity, the lack of formal statistical significance in the ≥70% stenosis subgroup (*P* = 0.078) should be interpreted with caution. Given that 171 of 225 patients (76%) in this subgroup already met the criteria for ischemia, the incremental predictive value of any biomarker—including FAI—is inherently constrained by the pre-test probability ceiling. Nevertheless, the consistency of the point estimate (OR 1.04) across stenosis categories suggests that the underlying biological relationship is preserved.

### Incremental value and clinical implications: NRI, IDI, and DCA

A critical component of biomarker validation is demonstrating not merely statistical independence but incremental clinical utility. In our analysis, the direct comparison of AUCs between the clinical model (0.707) and the combined FAI model (0.731) did not reach statistical significance (*P* = 0.087). This is not unexpected, as AUC is a relatively insensitive metric for detecting improvements in nested models, particularly when the baseline model already incorporates several strong predictors. Therefore, the term “significantly improves diagnostic performance” is not used in this manuscript; instead, we report the incremental value primarily through reclassification and decision-curve metrics. More importantly, the risk reclassification metrics provided compelling evidence of added value. The continuous NRI of 0.339 (*P* = 0.001) indicates a net improvement in risk classification when FAI is added to the clinical model. However, this continuous metric does not represent the proportion of patients reclassified; it reflects the overall improvement in the predicted probabilities of events and non-events. Similarly, the IDI of 0.024 (*P* = 0.002) confirms a net gain in both sensitivity and specificity across the entire risk spectrum.

The clinical relevance of these statistical improvements was further substantiated by DCA. Across a broad and clinically realistic range of threshold probabilities (25% to 75%), the FAI-integrated model yielded a higher net benefit than the clinical model alone. In practical terms, this suggests that using FAI to guide downstream testing or therapeutic intensification could help clinicians avoid unnecessary invasive procedures in patients with falsely positive anatomical findings, while identifying a subset of individuals with ischemia that is not fully explained by stenosis severity alone and may reflect a contribution from coronary inflammation.

### Comparison with existing literature

Our findings extend and refine the existing body of evidence linking FAI to adverse cardiovascular outcomes. The landmark CRISP-CT study established the prognostic value of elevated FAI for all-cause and cardiac mortality in patients undergoing clinically indicated CCTA (20). More recently, studies have explored the association between FAI and invasive physiological indices, reporting correlations with fractional flow reserve (FFR) and microvascular resistance (16). The present study advances the field by: (1) employing CT-FFR as a fully non-invasive, lesion-specific functional endpoint; (2) rigorously adjusting for EAT volume to isolate the contribution of perivascular inflammation; (3) providing a comprehensive assessment of risk reclassification (NRI/IDI) and clinical net benefit (DCA); and (4) systematically examining effect modification across clinically relevant subgroups. To our knowledge, this is among the first studies to demonstrate that FAI improves the identification of CT-FFR-defined ischemia with demonstrable clinical net benefit in an East Asian population.

### FAI vs. coronary artery calcium score: complementary imaging biomarkers

The present study did not directly compare the diagnostic value of FAI with the coronary artery calcium (CAC) score, and we acknowledge this as an important consideration. CAC score is an established marker of the total calcified atherosclerotic burden—an anatomical measure of disease extent—whereas FAI reflects the inflammatory activity of perivascular adipose tissue, a dynamic biological process that can be elevated even in the absence of coronary calcification. As the reviewer correctly noted, traditional cardiovascular risk factors have limited predictive value for myocardial ischemia; likewise, CAC score primarily quantifies plaque burden and does not capture lesion-specific hemodynamics or vascular inflammation. Our study therefore focused on whether FAI, as an inflammatory biomarker, provides incremental diagnostic value beyond conventional clinical variables and EAT volume, rather than comparing it with another anatomical measure of atherosclerosis. Nevertheless, we agree that head-to-head comparisons between FAI and CAC—and their potential complementary value—represent an important direction for future investigation, and we have acknowledged the absence of CAC data in our cohort as a limitation of this study.

### Study limitations

Several limitations warrant acknowledgment. First, this was a retrospective, single-center study, and residual confounding from selection bias or unmeasured factors cannot be excluded despite adjustment for multiple covariates.

Second, the reference standard for functional ischemia was CT-FFR rather than invasive FFR. Although CT-FFR correlates well with invasive FFR, it primarily reflects epicardial physiology and cannot distinguish microvascular dysfunction. In addition, other functional tests were performed only according to clinical indications and were not uniformly available as independent references; thus, verification bias cannot be excluded. Angina status was based on clinically documented symptoms, present in 175 (46.1%) patients; however, standardized symptom grading (e.g., the Canadian Cardiovascular Society classification) and differentiation between typical and atypical angina were not systematically captured.

Third, FAI analysis was limited to the proximal segments of the three major coronary arteries, and the primary exposure was the average of these values. Because elevated FAI can also occur in the setting of an unstable plaque confined to a single artery, averaging across vessels may theoretically dilute focal inflammatory signals. Although vessel-specific sensitivity analyses showed directionally consistent associations, with statistically significant independent associations for RCA FAI and LCX FAI, anatomically paired vessel-level validation was not possible because vessel identity of the lowest CT-FFR value was not retained. Future studies with vessel-matched analyses linking vessel-specific FAI to ischemia in the corresponding coronary territory are therefore needed.

Fourth, the FAI cutoff of −81.32 HU was data-driven and derived from the same cohort, making it exploratory and scanner- and protocol-dependent. FAI measurement reproducibility was not formally assessed, and the combined model was only internally validated by bootstrap, without external validation. Although the diagnostic accuracy of artificial intelligence-based CT-FFR algorithms has been previously validated (14), independent external validation data specifically for the FFRDoc software version remain limited.

Fifth, systemic inflammatory biomarkers such as hs-CRP were not available, so we could not evaluate whether FAI provides information independent of systemic inflammatory pathways.

In addition, coronary artery calcium (CAC) scoring was not systematically available in this retrospective cohort, precluding a direct comparison between FAI and CAC for the identification of functional ischemia; this represents a further limitation of the present study.

Finally, the number of ischemic events in some subgroups was small, particularly the 30%–75% stenosis group, limiting statistical power. These subgroup findings should therefore be interpreted cautiously.

### Future directions

Prospective, multicenter studies with blinded, core-laboratory FAI measurements and invasive FFR/IMR validation are warranted to confirm our observations. Furthermore, longitudinal studies are needed to determine whether therapeutic interventions that modulate perivascular inflammation—such as intensive statin therapy, anti-inflammatory agents (e.g., colchicine), or novel anti-diabetic drugs (e.g., SGLT2 inhibitors)—translate into measurable reductions in FAI and corresponding improvements in coronary physiological function. Such studies will help clarify whether the association between FAI and functional ischemia is mediated primarily by epicardial stenosis, coronary microvascular dysfunction, or a combination of both.

## Conclusion

In conclusion, coronary perivascular FAI is an independent and robust imaging biomarker for predicting CT-FFR-defined functional ischemia, with incremental value beyond traditional risk factors and EAT volume. The integration of FAI into clinical risk assessment significantly improves risk reclassification and provides incremental clinical net benefit. These findings position FAI as a promising non-invasive marker of coronary inflammation that may refine diagnostic and therapeutic decision-making in patients undergoing CCTA for suspected coronary artery disease, with the understanding that CT-FFR primarily reflects epicardial stenosis.

## Data Availability

The data analyzed in this study is subject to the following restrictions: The datasets generated and/or analyzed during this study are not publicly available due to patient privacy protection and institutional data security policies. Requests for access to the de-identified dataset may be directed to the corresponding author, subject to institutional review and approval. Requests to access these datasets should be directed to Min Jia, jiamin10086@126.com.
