# Supplementary material for: Perivascular fat attenuation index improves the identification of functional myocardial ischemia beyond traditional risk factors: a coronary CT angiography-derived fractional flow reserve study
Source: Front Cardiovasc Med. 2026 Oct 2;13:1950032. doi: 10.3389/fcvm.2026.1950032 (PMC13672025; doi:10.3389/fcvm.2026.1950032)
Supplement: Supplementary file 1 [file Table1.pdf]

Supplementary Materials

Supplementary Table S1. Prevalence of anatomical CAD, severe coronary stenosis, angina pectoris, and additional examinations in the overall cohort (N = 380)

Variable	Overall (N=380), n (%)
CCTA-defined anatomical CAD	356 (93.7)
Severe coronary stenosis ≥70%	225 (59.2)
Clinically documented angina pectoris	175 (46.1)
At least one additional functional or invasive examination	237 (62.4)
Exercise ECG or stress echocardiography	189 (49.7)
Myocardial perfusion imaging	83 (21.8)
Invasive coronary angiography	102 (26.8)
CT-FFR ≤0.80	194 (51.1)

Note. Data are presented as n (%). Anatomical CAD was defined as the presence of coronary atherosclerotic plaque on CCTA. Severe coronary stenosis was defined as ≥70% diameter stenosis in at least one major epicardial coronary artery. Angina pectoris was considered present only when angina, stable angina, or unstable angina was explicitly documented in the medical record. Additional examinations included exercise ECG or stress echocardiography, myocardial perfusion imaging, and invasive coronary angiography; some patients underwent more than one examination, and the individual examination categories were therefore not mutually exclusive. CT-FFR ≤ 0.80 indicates hemodynamic significance (functional ischemia). CAD, coronary artery disease; CCTA, coronary computed tomography angiography; CT-FFR, CT-derived fractional flow reserve; ECG, electrocardiography.

Supplementary Table S2. Sensitivity analyses replacing average FAI with individual-coronary FAI measurements

FAI measure	Unadjusted OR (95% CI)	P value	Adjusted OR (95% CI)	P value	Adjusted AUC
Average FAI	1.046 (1.015–	0.004	1.048 (1.015–	0.004	0.680

FAI measure	Unadjusted OR (95% CI)	P value	Adjusted OR (95% CI)	P value	Adjusted AUC
(reference)	1.079)		1.081)		
LAD FAI	1.012 (0.991–1.033)	0.259	1.016 (0.995–1.036)	0.130	0.661
LCX FAI	1.042 (1.013–1.071)	0.004	1.045 (1.014–1.077)	0.005	0.674
RCA FAI	1.046 (1.020–1.072)	<0.001	1.041 (1.014–1.068)	0.003	0.685

Note. ORs are reported per 1-HU increase (less negative FAI). Both unadjusted and multiply imputed adjusted models included 380 patients (194 events). Adjusted covariates were age, sex, smoking, alcohol consumption, hypertension, diabetes mellitus, systolic blood pressure, high-density lipoprotein cholesterol, and epicardial adipose tissue volume. Twenty imputed datasets were combined using Rubin's rules. AUC, area under the receiver operating characteristic curve; CI, confidence interval; FAI, fat attenuation index; LAD, left anterior descending artery; LCX, left circumflex artery; OR, odds ratio; RCA, right coronary artery.
